# Engineering efficient termination of bacteriophage T7 RNA polymerase transcription

**DOI:** 10.1093/g3journal/jkac070

**Published:** 2022-03-28

**Authors:** Diana G Calvopina-Chavez, Mikaela A Gardner, Joel S Griffitts

**Affiliations:** Department of Microbiology and Molecular Biology, Brigham Young University, Provo, UT 84602, USA

**Keywords:** T7 polymerase, transcription termination, synthetic terminators, T7 terminator, transcriptional pausing

## Abstract

The bacteriophage T7 expression system is one of the most prominent transcription systems used in biotechnology and molecular-level research. However, T7 RNA polymerase is prone to read-through transcription due to its high processivity. As a consequence, enforcing efficient transcriptional termination is difficult. The termination hairpin found natively in the T7 genome is adapted to be inefficient, exhibiting 62% termination efficiency in vivo and even lower efficiency in vitro. In this study, we engineered a series of sequences that outperform the efficiency of the native terminator hairpin. By embedding a previously discovered 8-nucleotide T7 polymerase pause sequence within a synthetic hairpin sequence, we observed in vivo termination efficiency of 91%; by joining 2 short sequences into a tandem 2-hairpin structure, termination efficiency was increased to 98% in vivo and 91% in vitro. This study also tests the ability of these engineered sequences to terminate transcription of the *Escherichia coli* RNA polymerase. Two out of 3 of the most successful T7 polymerase terminators also facilitated termination of the bacterial polymerase with around 99% efficiency.

## Introduction 

Protein expression systems derived from bacteriophage T7 are commonly used for high-level expression of recombinant proteins in engineered strains of *Escherichia coli* (Lebedeva *et al.* 1994; Conrad *et al.* 1996; Wycuff and Matthews 2000; Tabor 2001). In these systems, a protein-coding gene of interest is placed downstream of an 18-bp promoter that is specifically recognized by T7 RNA polymerase (T7RNAP; Rong *et al.* 1998). This construct is then introduced into an *E. coli* strain that has been modified to carry a copy of the T7RNAP gene on its chromosome. Expression of T7RNAP is typically controlled by a chemically inducible bacterial promoter, such as the *lac* promoter (Lebedeva *et al.* 1994; Rong *et al.* 1998). To enhance chemical control of the system, a *lac* operator may also be installed downstream of the T7 promoter on the expression plasmid (Studier and Moffatt 1986; Dubendorf and Studier 1991; Studier 1991). T7RNAP transcribes DNA at a higher rate than *E. coli* RNA polymerase (EcRNAP; Golomb and Chamberlin 1974; Iost *et al.* 1992), but its high processivity can result in read-through transcription and even circumnavigation of the entire expression plasmid. T7RNAP-generated transcripts are therefore often much longer than necessary, resulting in overexpression of unwanted proteins (McAllister *et al.* 1981; Tabor and Richardson 1985).

The native T7 terminator (T7nat) is found in the T7 phage genome between genes 10 and 11 (Dunn and Studier 1983), and its sequence and predicted structure are given in Table 1 and Supplementary Fig. 1, respectively. This terminator features a 13-bp stem with 5 G-U base pairs, a 6-nt loop, and a 3′ poly-U tract. T7nat is tuned to be inefficient in its native context, allowing upstream genes to be expressed at higher levels than the more modestly expressed downstream genes (Dunn and Studier 1983). Read-through transcription is thus built into the T7 genetic program to the benefit of the phage. In most protein expression plasmids, T7nat is found within a larger sequence context that promotes stronger termination efficiency than is accommodated by the core hairpin sequence described above (Kwon and Kang 1999; Song and Kang 2001). In one study, termination efficiency was found to decrease by half when the 100-bp sequence upstream of the terminator hairpin was omitted (Yoo and Kang 1996). This has prompted a search for more compact sequences facilitating stronger T7RNAP termination. In one such study, a modified T7 terminator sequence employing a more structurally favorable UUCG loop, as well as replacement of certain G-U base pairs with G-C base pairs, yielded a 40% improvement in termination efficiency in vitro (Mairhofer *et al.* 2015).

**Table 1.** DNA sequence of engineered terminators.

Name	Purpose	DNA sequence^*a*,*b*^
T7nat	Wild-type sequence	AACCCCTTGGGGCCTCTAAACGGGTCTTGAGGGGTTTTTTTT
T7mod	Stronger stem, shorter loop	AACCCTGCGAGGCCTC ttcg GAGGTCTCGCAGGGTT TTTTTT
T7pause	Dual pause sites, no hairpin	CtatctgttatctgttC
T7hyb1	T7mod with single pause	AAACAGATAGGCCCTC ttcg GAGGGCCtatctgttT TTTTTT
T7hyb2	Short variant of T7hyb1	AACAGATAGGCCTC ttcg GAGGCCtatctgtt TTTTTT
T7hyb3	T7hyb2, higher GC%	AACAGATAGGCCGC ttcg GCGGCCtatctgtt TTTTTT
T7hyb4	T7hyb3 altered GC sequence	AACAGATAGCCGCG ttcg CGCGGCtatctgtt TTTTTT
T7hyb5	Short variant of T7hyb3	AGATAGGCCGC ttcg GCGGCCtatctgt tTTTTTT
T7hyb6	Dual-pause hairpin	AGATAACAGATAC ttcg Gtatctgttatct gttTTTTTT
T7hyb7	T7hyb6, pause sites scrambled—variant 1	AAGATAAGCAATC ttcg GATTGCTTATCTT GTTTTTTTT
T7hyb8	T7hyb6, pause sites scrambled—variant 2	TAAAGAATAAACC ttcg GGTTTATTCTTTA GTTTTTTTT
T7hyb9	Fusion: T7hyb4-T7hyb6	AACAGATAGCCGCG ttcg CGCGGCtatctgtt TTTTTT CAGATAACAGATAC ttcg Gtatctgttatctg ttTTTTTT
T7hyb10	Fusion: T7hyb6-T7hyb4	AGATAACAGATAC ttcg Gtatctgttatct gttTTTTTTc AACAGATAGCCGCG ttcg CGCGGCtatctgtt TTTTTT

a Underlined sequence indicates designed stem structures

b Lower case indicates UUCG loop structures and TATCTGTT single- or double-pause sites

In addition to hairpin-forming (Class I) T7RNAP terminators, unstructured and sequence-dependent (Class II) termination signals have been identified (Macdonald *et al.* 1994; Du *et al.* 2012), primarily through in vitro assays on nonphage DNA templates. One such sequence (TATCTGTT) derived from vesicular stomatitis virus (VSV), stimulates T7RNAP pausing, which potentiates termination under in vitro conditions (Whelan *et al.* 1995; Lyakhov *et al.* 1998; Barr and Wertz 2001). For reasons not well understood, the VSV pause sequence is not effective in vivo (Du *et al.* 2009, 2012). This may be due to differences in transcription rate, DNA topology, or other unknown cellular factors.

In previous work, researchers have sought for more efficient T7RNAP terminators that could allow for higher protein expression in vivo with lower metabolic burden, or for insulation of expression modules within multigene expression plasmids (Du *et al.* 2009, 2012; Mairhofer *et al.* 2015). Strategies included the use of tandem Class I terminators, tandem Class II terminators, and tandem cassettes bearing both Class I and Class II terminators (Du *et al.* 2009, 2012; Mairhofer *et al.* 2015). In this report, we explore whether the integration of a Class II pause sequence within the hairpin of a Class I sequence can enhance termination efficiency beyond what is observed in either type alone. The compact and efficient properties of such engineered terminators could be of some value for enhancing protein expression, insulating distinct expression modules, and building customized and modular T7 expression plasmids for diverse purposes.

## Materials and methods

### Bacterial strains, plasmids, and media conditions

All plasmids and strains used in this study are summarized in Supplementary Tables 1 and 2. For cloning purposes, all plasmids were first transformed into DH5α *E. coli* cells. Plasmids were then extracted and transformed into MG1655 WT *E. coli* cells for measuring transcription termination by fluorescence (see below). Cultures were grown at 37°C in Luria broth medium supplemented with chloramphenicol (30 µg/ml) and ampicillin (100 µg/ml) or only ampicillin (100 µg/ml) as appropriate.

### Plasmid construction for each terminator

A unique pair of oligonucleotides was hybridized to create the inserts containing each terminator sequence. Hybridized oligos were designed to have sticky ends complementary to *Bam*HI and *Kpn*I. Oligo sequences are detailed in Supplementary Table 3. For oligo hybridization, TEN buffer (5 mM Tris pH 8.0, 0.5 mM EDTA pH 8.0, 50 mM NaCl) was prepared and top and bottom oligos were added to a final concentration of 10 µM each. The mixture was then brought to: 95°C for 1 min, 90°C for 15 min, 75°C for 20 min, 70°C for 20 min, 65°C for 20 min, 60°C for 20 min, 55°C for 20 min, and then held at 4°C. Parent plasmid pJG1113 containing GFP-*Bam*HI-*Kpn*I-RFP was digested with *Bam*HI and *Kpn*I, and inserts with each terminator were ligated.

### RNAP transcriptional termination measurements in vivo

Plasmids were transformed into *E. coli* MG1655 and reporter gene expression was measured after culturing strains for 8 h at 37°C. To measure T7 RNAP termination, basal level expression of T7RNAP (with no arabinose added) was relied upon in these experiments. OD_600_ measurements were taken to adjust cell density in each culture before fluorescence measurements. After subtracting background fluorescence (based on a control strain lacking T7RNAP), the red/green ratio from the no-terminator control strain allowed for normalization to 1. This was compared to all red/green ratios from the terminator constructs to determine % termination efficiency. For measurements of *E. coli* RNAP termination, similar conditions were used, except cultures were supplemented with 0.3 mM IPTG.

### In vitro transcription reactions

Linear terminator containing DNA templates were amplified from corresponding plasmids (see Supplementary Table 1). The forward primer (oDC81) had the T7 promoter sequence appended, and it hybridized around 100 bp upstream of the terminator; the reverse primer (oDC82) was designed to hybridize around 100 bp downstream of the terminator. After DNA amplification and purification, transcription reactions were carried out in a volume of 20 µl of RNase free water, 1x RNase buffer, 0.5 mM NTPs, 1 U/µl RNase inhibitor, 1 µg of linear DNA template, 2U/µl T7RNAP (NEB M0251S). Reactions were incubated at 37°C for 3 h. They were then placed at 75°C for 10 min to inactivate the enzyme. Reactions were treated with DNase I (1U/µl) at 37°C for 20 min. RNA products were concentrated using the RNA Clean & Concentrator kit from Zymo (R1015), and 15 µl of the sample was resolved in an 8% polyacrylamide gel in the presence of 7M Urea and 3 µg/ml ethidium bromide (Supplementary Fig. 2). RNA products were visualized in a UV gel imager. Termination efficiency percentages were obtained by calculating the ratio of the integrated density of the terminated (lower molecular weight) band over the integrated density of the entire lane. Integrated densities were obtained with ImageJ software.

## Results and discussion

### Assay for measuring T7RNAP termination in vivo

**Fig. 1.**
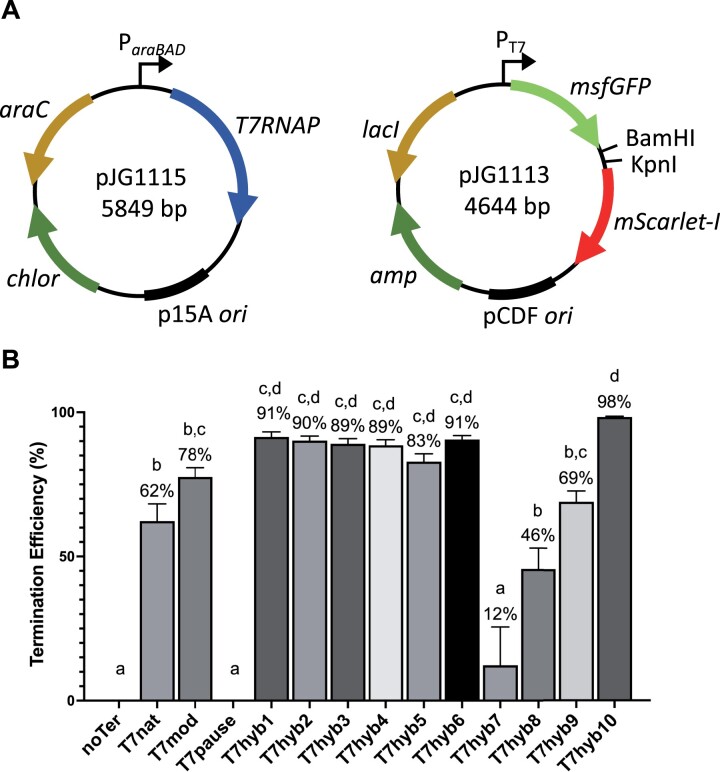
In vivo termination efficiency of designed T7 transcriptional terminators. a) A 2-plasmid system allows T7RNAP expression from an arabinose-inducible promoter and monitoring of T7RNAP-dependent transcription from a T7 promoter driving green (*msfGFP*) and red (*mScarlet-I*) fluorescent proteins. b) Error bars represent standard deviation from the mean. Different letters denote statistically significant differences (*P* < 0.05) according to a Tukey multiple comparison test.

A 2-plasmid system was used to monitor T7RNAP termination efficiency in vivo, where plasmid pJG1115 expresses the gene for T7RNAP using the L-arabinose-inducible (P_araBAD_) promoter. The second plasmid (pJG1113) contains a bicistronic cassette encoding a green fluorescent protein (msfGFP; hereafter referred to as GFP) and a red fluorescent protein (mScarlet-I; hereafter referred to as RFP) in which the restriction sites *Bam*HI and *Kpn*I are used to ligate terminator candidates between the 2 fluorescent protein reporters. This biscistronic cassette is expressed using the T7 promoter (P_T7_; see Fig. 1a for complete plasmid maps; see Supplemental Material file for complete DNA sequence of parent plasmids used in this study). GFP/RFP ratiometric analysis allows T7RNAP termination efficiency to be calculated, using control strains with no T7RNAP and with no terminator sequence (see *Materials* *and* *Methods*).

### Design of synthetic terminators

T7nat was used as a reference sequence throughout this study. A potentially more effective variant of T7nat, termed T7mod, was inspired by the UUCG loop-containing sequence described above (Mairhofer *et al.* 2015). These are both Class I terminators. As a Class II terminator sequence, we employed a known pause site (the VSV sequence described above: TATCTGTT), and this was inserted as 2 copies that overlap by 1 base pair (T7pause; see Table 1). In remaining test sequences (T7hybX, where X is 1-10), Class I and Class II elements were combined, with pause sequences embedded in the poly-U-proximal segment of the terminator stem. T7hyb1-5 contain a single pause site and differ in stem length and stem %GC; T7hyb6 contains 2 slightly overlapping pause sites; T7hyb7 and T7hyb8 mimic T7hyb6, forming perfect hairpins but with the double-pause sequences being scrambled such that overall base composition was not altered. Tandem double-hairpins were also tested (T7hyb9 and T7hyb10). These designs allowed us to evaluate the influence of several structural and sequence parameters, including the embedded pause sequences. These sequences are summarized in Table 1, and Supplementary Table 3 details the synthetic oligonucleotides used to clone these sequences.

### In vivo and in vitro performance of terminator designs

T7nat, T7mod, T7pause, and T7hyb1-10 were ligated into the GFP-RFP reporter plasmid and assayed for in vivo termination efficiency (see Supplementary Tables 1 and 2 for plasmids and strains details). The results of these tests are given in Fig. 1b. T7nat exhibits 62% termination efficiency, with T7mod having modestly increased activity. T7pause shows no detectable activity in this assay. This is not surprising as previous reports have shown that Class II pause sites facilitate termination much more efficiently in vitro than in vivo (Du *et al.* 2009; 2012). Where a single pause site is incorporated in the hairpin (T7hyb1-5), efficiency varies from 83% to 91%, with the short-stem variant (T7hyb5) showing the lowest efficiency. The double-pause design (T7hyb6) provides 91% efficiency despite the much lower GC content in the stem. To test whether the TATCTGTT pause sequences in T7hyb6 are critical, they were scrambled, resulting in molecules that would fold similarly, with identical %GC to T7hyb6. We predicted that these scrambled variants (T7hyb7 and T7hyb8) would exhibit lower termination efficiency. They indeed showed much lower termination efficiency (12% and 46%, respectively), indicating that nucleotide order in these pause sequences strongly contributes to the performance of T7hyb6, even though the double-pause sequence on its own (T7pause) is ineffective. Finally, tandem double-hairpins were tested. The efficiency of T7hyb9 (tandem T7hyb4-T7hyb6) was lower than either single hairpin alone, while T7hyb10 (tandem T7hyb6-T7hyb4) was the most efficient of the whole set, at 98%. Why 2 single-hairpin elements joined in 2 possible orientations leads to such different termination efficiencies is unclear.

**Fig. 2.**
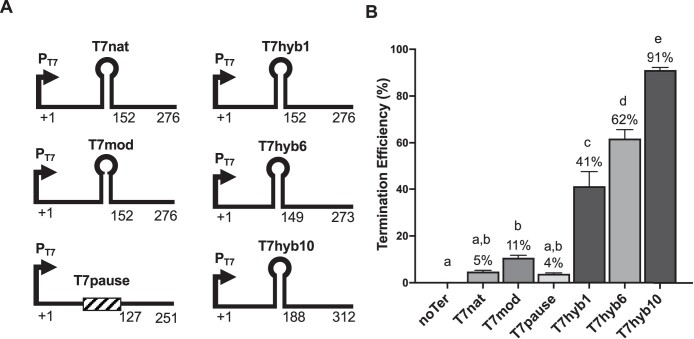
Performance of selected terminators in vitro. a) Diagram of transcription templates for in vitro testing using recombinant T7RNAP. The 3 numbers underneath each diagram show the transcription initiation site, the predicted site of termination, and the length of the run-off product. Hairpin symbols represent the cloned T7 terminators. b) In vitro termination efficiency of selected terminators. The error bars represent standard deviation from the mean. Different letters denote statistically significant differences (*P* < 0.05) according to a Tukey multiple comparison test.

In vitro tests were carried out on a subset of the terminator sequences using recombinant T7RNAP (New England Biolabs) and PCR-generated DNA templates. Transcription products (size range: 127–312 nt) were resolved on polyacrylamide-urea gels and stained with ethidium bromide. Ratiometric analysis of terminated and run-off products was performed using Image J (see Supplementary Table 4 for integrated density values obtained from ImageJ for ratiometric analysis). In these tests, the relative efficiencies of terminator sequences reflected those determined in vivo, though calculated in vitro values were considerably lower (see Fig. 2). Notably, however, the T7pause sequence did register a detectable amount of termination, and T7hyb6 (with its double pause) showed significantly enhanced termination over T7hyb1 (with only a single pause), while T7hyb1 and T7hyb6 were equivalently effective in vivo. We interpret this to mean that the pause sites, either alone or embedded in a hairpin, contribute more to termination efficiency in vitro than in vivo. This interpretation is supported by previous work (Du *et al.* 2012). As in the in vivo analysis, T7hyb10 was the best-performing design.

### Terminator activity for the *E. coli* bacterial RNAP

**Fig. 3.**
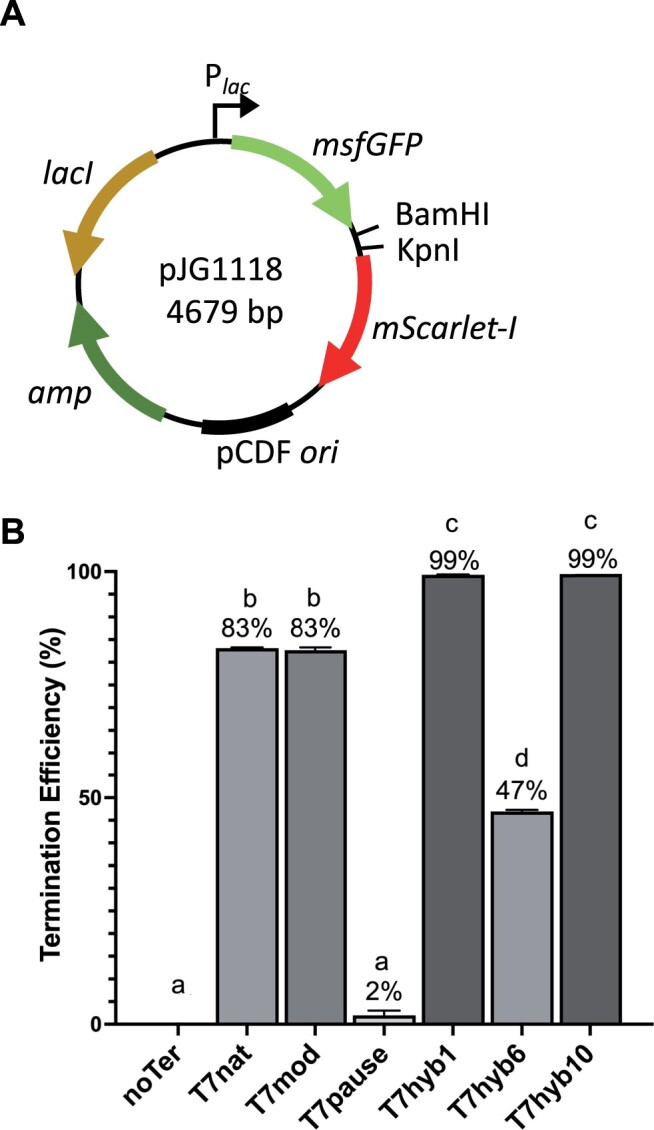
In vivo performance of selected terminators with the bacterial polymerase EcRNAP. a) Terminator sequences were placed between fluorescent protein-encoding genes located downstream of the *lac* promoter, using *Bam*HI and *Kpn*I restriction sites. Plasmids were transformed into *E. coli* MG1655 and reporter gene expression was used to assess terminator efficiency. b) Bar chart of the mean termination efficiency of engineered terminators for EcRNAP. Error bars represent standard deviation from the mean. Different letters denote statistically significant differences (*P* < 0.05) according to a Tukey multiple comparison test.

To determine whether these synthetic terminators would be more broadly applicable in *E. coli*-based expression systems, they were tested in the context of the native bacterial polymerase (EcRNAP). To do this, pJG1113 (see Fig. 1a) was modified to use the *lac* promoter to drive the GFP-RFP cassette (pJG1118; see Fig. 3a). The same terminator subset used in the in vitro analysis was investigated here. As shown in Fig. 3b, T7hyb1 and T7hyb10 gave outstanding termination efficiencies (99%) for EcRNAP in vivo. For all the data taken together, the tandem hairpin T7hyb10 sequence is an excellent all-around termination signal for RNA polymerases from both T7 (98%) and *E. coli* (99%). For a simpler single-hairpin alternative, T7hyb1 performs best, with in vivo efficiency values of 91% for the phage polymerase and 99% for the bacterial polymerase.

In previous attempts to make stronger T7 terminators, researchers have explored combinations of VSV pause sequences and Class I transcriptional terminators such as T7nat and T7mod. One such combination exhibited higher efficiency than T7nat alone, leading to higher protein expression due to lower metabolic burden on expressing cells (Mairhofer *et al.* 2015); however, this was a considerably long sequence (183 bp). In another study, researchers used tandem repeats of the VSV pause sequence to create shorter terminators that were efficient in vitro but not in vivo (Du *et al.* 2009; 2012). We have developed novel chimeric and compact terminators bearing Class II pause sequences within Class I-type hairpins, which exhibit strong termination efficiency in vivo and in vitro. Combining 2 of these chimeric sequences in tandem (with a total length of 78 base pairs) results in nearly 100% in vivo termination efficiency in the context of either the EcRNAP or T7RNAP.

## Data availability

Bacterial strains and plasmids are available upon request. The data and reagents underlying this article are available within the article and in the supplemental material section.

Supplemental material is available at *G3* online.

## Funding

Financial support for this work was provided by the National Institutes of Health (NIH) grant 1R15GM132852-01.

## Conflicts of interest

None declared.

## Supplementary Material

jkac070_Supplementary_Figure_S1

jkac070_Supplementary_Figure_S2

jkac070_Supplementary_Tables_and_Figure_Legends
