# Supplementary material for: Engineering efficient termination of bacteriophage T7 RNA polymerase transcription
Source: G3 (Bethesda). 2022 Mar 28;12(6):jkac070. doi: 10.1093/g3journal/jkac070 (PMC9157156; doi:10.1093/g3journal/jkac070)
Supplement: jkac070_Supplementary_Figure_S1 [file jkac070_supplementary_figure_s1.pdf]

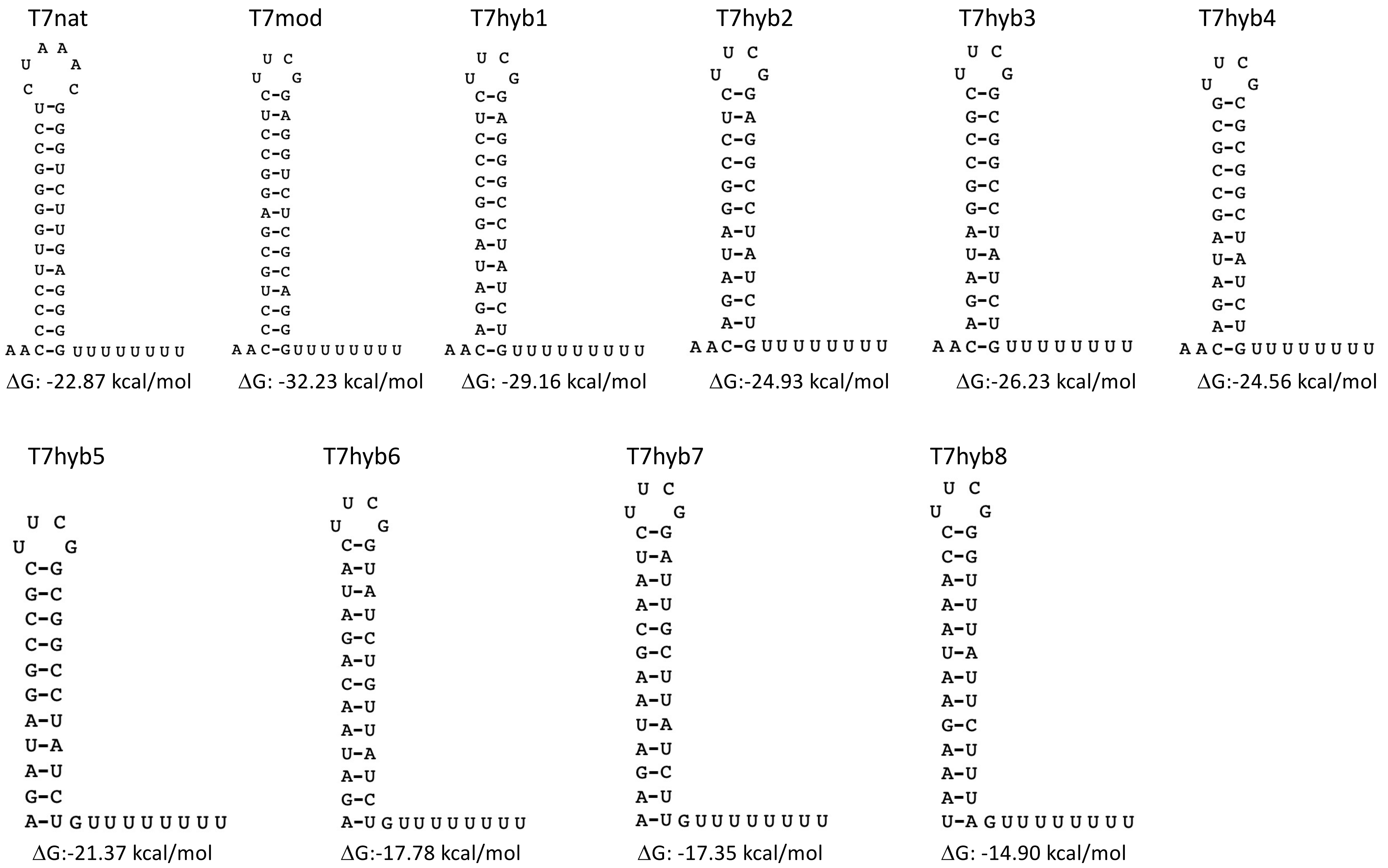
T7nat
ΔG: -22.87 kcal/mol
T7mod
ΔG: -32.23 kcal/mol
T7hyb1
ΔG: -29.16 kcal/mol
T7hyb2
ΔG:-24.93 kcal/mol
T7hyb3
ΔG:-26.23 kcal/mol
T7hyb4
ΔG:-24.56 kcal/mol
T7hyb5
ΔG:-21.37 kcal/mol
T7hyb6
ΔG:-17.78 kcal/mol
T7hyb7
ΔG: -17.35 kcal/mol
T7hyb8
ΔG: -14.90 kcal/mol
