# Supplementary figures and images for: Engineering efficient termination of bacteriophage T7 RNA polymerase transcription
Source: G3 (Bethesda). 2022 Mar 28;12(6):jkac070. doi: 10.1093/g3journal/jkac070 (PMC9157156; doi:10.1093/g3journal/jkac070)

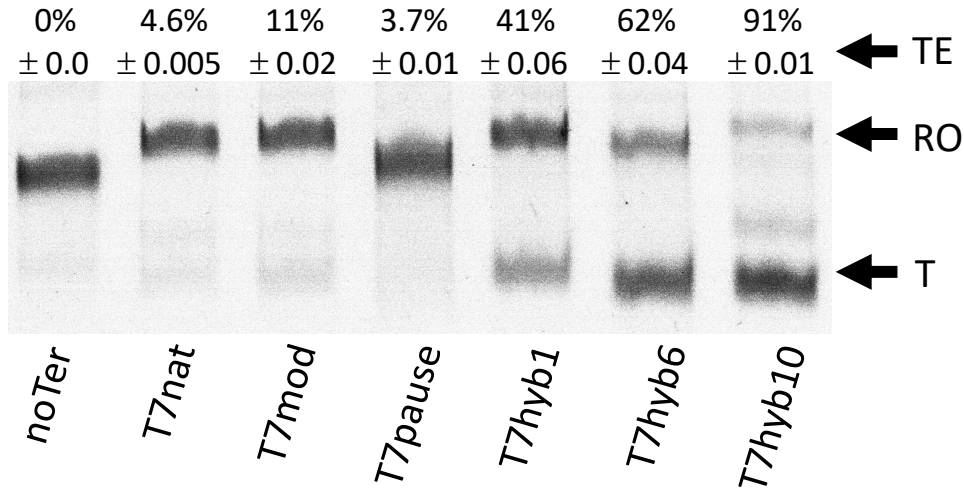

Supplement: jkac070_Supplementary_Figure_S2 [file jkac070_supplementary_figure_s2.pdf]
