# Supplementary material for: Engineering efficient termination of bacteriophage T7 RNA polymerase transcription
Source: G3 (Bethesda). 2022 Mar 28;12(6):jkac070. doi: 10.1093/g3journal/jkac070 (PMC9157156; doi:10.1093/g3journal/jkac070)
Supplement: jkac070_Supplementary_Tables_and_Figure_Legends [file jkac070_supplementary_tables_and_figure_legends.docx]

**SUPPLEMENTAL FIGURE LEGENDS AND TABLES**

**Figure S1.** Predicted RNA structures and free energy (ΔG) values of single hairpin terminators tested in this study.

**Figure S2.** To calculate in vitro termination efficiency, transcripts from in vitro reactions were resolved in an 8% polyacrylamide gel in the presence of 7M Urea and 3 µg/ml ethidium bromide in triplicates. The higher molecular weight bands represent the run-off (RO) transcripts and the lower molecular weight bands represent the terminated (T) RNA product. Termination efficiency (TE) was calculated by comparing the band densities using ImageJ.

**Table S1. Plasmids**

| Plasmid name | *E. coli* background | Parent plasmid | Purpose/Insert |
| --- | --- | --- | --- |
| pED022 | DH5α |  | No P_T7_-GFP/RFP, no terminator |
| pJG1113 | DH5α |  | P_T7_-GFP/RFP, no terminator |
| pJG727 | DH5α |  | No T7RNAP control |
| pJG1115 | DH5α | pJG727 | T7RNAP |
| pJG1124 | DH5α | pJG1113 | T7nat |
| pJG1126 | DH5α | pJG1113 | T7mod |
| pDC42 | DH5α | pJG1113 | T7pause |
| pJG1128 | DH5α | pJG1113 | T7hyb1 |
| pJG1130 | DH5α | pJG1113 | T7hyb2 |
| pJG1131 | DH5α | pJG1113 | T7hyb3 |
| pJG1133 | DH5α | pJG1113 | T7hyb4 |
| pJG1135 | DH5α | pJG1113 | T7hyb5 |
| pJG1137 | DH5α | pJG1113 | T7hyb6 |
| pDC38 | DH5α | pJG1113 | T7hyb7 |
| pDC39 | DH5α | pJG1113 | T7hyb8 |
| pDC40 | DH5α | pJG1113 | T7hyb9 |
| pDC41 | DH5α | pJG1113 | T7hyb10 |
| pJG1118 | DH5α | pJG1113 | P_lac_-GFP/RFP, no terminator |
| pDC44 | DH5α | pJG1118 | T7nat |
| pDC45 | DH5α | pJG1118 | T7mod |
| pDC49 | DH5α | pJG1118 | T7pause |
| pDC46 | DH5α | pJG1118 | T7hyb1 |
| pDC47 | DH5α | pJG1118 | T7hyb6 |
| pDC48 | DH5α | pJG1118 | T7hyb10 |

**Table S2. Bacterial Strains**

| Strain name | *E. coli* background | Plasmid{s} | Purpose/Figure |
| --- | --- | --- | --- |
| DC125 | MG1655 | pJG727+pED022 | No T7RNAP, No P_T7_-GFP/RFP, no terminator; Fig. 1 |
| DC126 | MG1655 | pJG727+pJG1113 | No T7RNAP; Fig. 1 |
| DC127 | MG1655 | pJG1115+pJG1113 | T7RNAP, no terminator; Fig. 1 |
| DC128 | MG1655 | pJG1115+pJG1124 | T7RNAP, T7nat; Fig. 1 |
| DC129 | MG1655 | pJG1115+pJG1126 | T7RNAP, T7mod; Fig. 1 |
| DC140 | MG1655 | pJG1115+pDC42 | T7RNAP, T7pause; Fig. 1 |
| DC130 | MG1655 | pJG1115+pJG1128 | T7RNAP, T7hyb1; Fig. 1 |
| DC131 | MG1655 | pJG1115+pJG1130 | T7RNAP, T7hyb2; Fig. 1 |
| DC132 | MG1655 | pJG1115+pJG1131 | T7RNAP, T7hyb3; Fig. 1 |
| DC133 | MG1655 | pJG1115+pJG1133 | T7RNAP, T7hyb4; Fig. 1 |
| DC134 | MG1655 | pJG1115+pJG1135 | T7RNAP, T7hyb5; Fig. 1 |
| DC135 | MG1655 | pJG1115+pJG1137 | T7RNAP, T7hyb6; Fig. 1 |
| DC136 | MG1655 | pJG1115+pDC38 | T7RNAP, T7hyb7; Fig. 1 |
| DC137 | MG1655 | pJG1115+pDC39 | T7RNAP, T7hyb8; Fig. 1 |
| DC138 | MG1655 | pJG1115+pDC40 | T7RNAP, T7hyb9; Fig. 1 |
| DC139 | MG1655 | pJG1115+pDC41 | T7RNAP, T7hyb10; Fig. 1 |
| DC141 | MG1655 | pED022 | No P_lac_-GFP/RFP, no terminator; Fig. 3 |
| DC142 | MG1655 | pJG1118 | No terminator; Fig. 3 |
| DC143 | MG1655 | pDC44 | T7nat; Fig. 3 |
| DC144 | MG1655 | pDC45 | T7mod; Fig. 3 |
| DC148 | MG1655 | pDC49 | T7pause; Fig. 3 |
| DC145 | MG1655 | pDC46 | T7hyb1; Fig. 3 |
| DC146 | MG1655 | pDC47 | T7hyb6; Fig. 3 |
| DC147 | MG1655 | pDC48 | T7hyb10; Fig. 3 |

**Table S3. Oligonucleotides for hybridizing terminator inserts**

| Oligo name | Sequence | Terminator |
| --- | --- | --- |
| 2008 | gatcAACCCCTTGGGGCCTctaaacGGGTCTTGAGGGGTTTTTTTTgtac | T7nat |
| 2009 | AAAAAAAACCCCTCAAGACCCgtttagAGGCCCCAAGGGGTT | T7nat |
| 2010 | gatcAACCCTGCGAGGCCTCttcgGAGGTCTCGCAGGGTTTTTTTTgtac | T7mod |
| 2011 | AAAAAAAACCCTGCGAGACCTCcgaaGAGGCCTCGCAGGGTT | T7mod |
| oDC56 | gatcCTATCTGTTATCTGTTCgtac | T7pause |
| oDC57 | GAACAGATAACAGATAG | T7pause |
| 2012 | gatcAAACAGATAGGCCCTCttcgGAGGGCCTATCTGTTTTTTTTTgtac | T7hyb1 |
| 2013 | AAAAAAAAACAGATAGGCCCTCcgaaGAGGGCCTATCTGTTT | T7hyb1 |
| 2014 | gatcAACAGATAGGCCTCttcgGAGGCCTATCTGTTTTTTTTgtac | T7hyb2 |
| 2015 | AAAAAAAACAGATAGGCCTCcgaaGAGGCCTATCTGTT | T7hyb2 |
| 2039 | gatcAACAGATAGGCCGCttcgGCGGCCTATCTGTTTTTTTTgtac | T7hyb3 |
| 2040 | AAAAAAAACAGATAGGCCGCcgaaGCGGCCTATCTGTT | T7hyb3 |
| 2041 | gatcAACAGATAGCCGCGttcgCGCGGCTATCTGTTTTTTTTgtac | T7hyb4 |
| 2042 | AAAAAAAACAGATAGCCGCGcgaaCGCGGCTATCTGTT | T7hyb4 |
| 2043 | gatcAGATAGGCCGCttcgGCGGCCTATCTGTTTTTTTTgtac | T7hyb5 |
| 2044 | AAAAAAAACAGATAGGCCGCcgaaGCGGCCTATCT | T7hyb5 |
| 2045 | gatcAGATAACAGATACttcgGTATCTGTTATCTGTTTTTTTTgtac | T7hyb6 |
| 2046 | AAAAAAAACAGATAACAGATACcgaaGTATCTGTTATCT | T7hyb6 |
| oDC48 | gatcAAGATAAGCAATCttcgGATTGCTTATCTTGTTTTTTTTgtac | T7hyb7 |
| oDC49 | AAAAAAAACAAGATAAGCAATCcgaaGATTGCTTATCTT | T7hyb7 |
| oDC50 | gatcTAAAGAATAAACCttcgGGTTTATTCTTTAGTTTTTTTTgtac | T7hyb8 |
| oDC51 | AAAAAAAACTAAAGAATAAACCcgaaGGTTTATTCTTTA | T7hyb8 |
| oDC52 | gatcAACAGATAGCCGCGttcgCGCGGCTATCTGTTTTTTTTCAGATAACAGATACttcgGTATCTGTTATCTGTTTTTTTTgtac | T7hyb9 |
| oDC53 | AAAAAAAACAGATAACAGATACcgaaGTATCTGTTATCTG AAAAAAAACAGATAGCCGCGcgaaCGCGGCTATCTGTT | T7hyb9 |
| oDC54 | gatcAGATAACAGATACttcgGTATCTGTTATCTGTTTTTTTTcAACAGATAGCCGCGttcgCGCGGCTATCTGTTTTTTTTgtac | T7hyb10 |
| oDC55 | AAAAAAAACAGATAGCCGCGcgaaCGCGGCTATCTGTTg AAAAAAAACAGATAACAGATACcgaaGTATCTGTTATCT | T7hyb10 |

**Table S4. Termination efficiency calculation for in vitro reactions in triplicates.**

| Lane # | Normalized Termination efficiency (%) |
| --- | --- |
| 1 | 0.0% ± 0.0 |
| 2 | 4.6% ± 0.005 |
| 3 | 11% ± 0.02 |
| 4 | 3.7% ± 0.01 |
| 5 | 41% ± 0.06 |
| 6 | 62% ± 0.04 |
| 7 | 91% ± 0.01 |

**Plasmid Sequences**

**pJG1115**

*araC* is located in position 57-935 bp

T7RNAP ORF is located at 1279-3930 bp

Gene that confers Chloramphenicol resistance is located at position 4119-4778 bp

p15A *ori* is located in 5031-5576 bp

CAGCTTGCCCACGTAGACCGACccgtcaagccgtcaattgtctgattcgttaccaaTTAtgacaacttgacggctacatcattcactttttcttcacaaccggcacgaaactcgctcgggctggccccggtgcattttttaaatactcgcgagaaatagagttgatcgtcaaaaccaacattgcgaccgacggtggcgataggcatccgggtagtgctcaaaagcagcttcgcctgactaatgcgttggtcctcgcgccagcttaagacgctaatccctaactgctggcggaaaagatgtgacagacgcgacggcgacaagcaaacatgctgtgcgacgctggcgatatcaaaattgctgtctgccaggtgatcgctgatgtactgacaagcctcgcgtacccgattatccatcggtggatggagcgactcgttaatcgcttccatgcgccgcagtaacaattgctcaagcagatttatcgccagcagctccgaatagcgcccttccccttgcccggcgttaatgatttgcccaaacaggtcgctgaaatgcggctggtgcgcttcatccgggcgaaagaaacccgtattggcaaatattgacggccagttaagccattcatgccagtaggcgcgcggacgaaagtaaacccactggtgataccattcgcgagcctccggatgacgaccgtagtgatgaatctctcctggcgggaacagcaaaatatcacccggtcggcagacaaattctcgtccctgatttttcaccaccccctgaccgcgaatggtgagattgagaatataacctttcattcccagcggtcggtcgataaaaaaatcgagataaccgttggcctcaatcggcgttaaacccgccaccagatgggcgttaaacgagtatcccggcagcaggggatcattttgcgcttcagcCATacttttcatactcccaccattcagagaagaaaccaattgtccatattgcatcagacattgccgtcactgcgtcttttactggctcttctcgctaacccaaccggtaaccccgcttattaaaagcattctgtaacaaagcgggaccaaagccatgacaaaaacgcgtaacaaaagtgtctataatcacggcagaaaagtccacattgattatttgcacggcgtcacactttgctatgccatagcatttttatccataagattagcggatcctacctgacgctttttatcgcaactctctactgtttctccatacccgtttGGTACCaccTCTAGAgGtatacatATGaacacgattaacatcgctaagaacgacttctctgacatcgaactggctgctatcccgttcaacactctggctgaccattacggtgagcgtttagctcgcgaacagttggcccttgagcatgagtcttacgagatgggtgaagcacgcttccgcaagatgtttgagcgtcaacttaaagctggtgaggttgcggataacgctgccgccaagcctctcatcactaccctactccctaagatgattgcacgcatcaacgactggtttgaggaagtgaaagctaagcgcggcaagcgcccgacagccttccagttcctgcaagaaatcaagccggaagccgtagcgtacatcaccattaagaccactctggcttgcctaaccagtgctgacaatacaaccgttcaggctgtagcaagcgcaatcggtcgggccattgaggacgaggctcgcttcggtcgtatccgtgaccttgaagctaagcacttcaagaaaaacgttgaggaacaactcaacaagcgcgtagggcacgtctacaagaaagcatttatgcaagttgtcgaggctgacatgctctctaagggtctactcggtggcgaggcgtggtcttcgtggcataaggaagactctattcatgtaggagtacgctgcatcgagatgctcattgagtcaaccggaatggttagcttacaccgccaaaatgctggcgtagtaggtcaagactctgagactatcgaactcgcacctgaatacgctgaggctatcgcaacccgtgcaggtgcgctggctggcatctctccgatgttccaaccttgcgtagttcctcctaagccgtggactggcattactggtggtggctattgggctaacggtcgtcgtcctctggcgctggtgcgtactcacagtaagaaagcactgatgcgctacgaagacgtttacatgcctgaggtgtacaaagcgattaacattgcgcaaaacaccgcatggaaaatcaacaagaaagtcctagcggtcgccaacgtaatcaccaagtggaagcattgtccggtcgaggacatccctgcgattgagcgtgaagaactcccgatgaaaccggaagacatcgacatgaatcctgaggctctcaccgcgtggaaacgtgctgccgctgctgtgtaccgcaaggacaaggctcgcaagtctcgccgtatcagccttgagttcatgcttgagcaagccaataagtttgctaaccataaggccatctggttcccttacaacatggactggcgcggtcgtgtttacgctgtgtcaatgttcaacccgcaaggtaacgatatgaccaaaggactgcttacgctggcgaaaggtaaaccaatcggtaaggaaggttactactggctgaaaatccacggtgcaaactgtgcgggtgtcgataaggttccgttccctgagcgcatcaagttcattgaggaaaaccacgagaacatcatggcttgcgctaagtctccactggagaacacttggtgggctgagcaagattctccgttctgcttccttgcgttctgctttgagtacgctggggtacagcaccacggcctgagctataactgctcccttccgctggcgtttgacgggtcttgctctggcatccagcacttctccgcgatgctccgagatgaggtaggtggtcgcgcggttaacttgcttcctagtgaaaccgttcaggacatctacgggattgttgctaagaaagtcaacgagattctacaagcagacgcaatcaatgggaccgataacgaagtagttaccgtgaccgatgagaacactggtgaaatctctgagaaagtcaagctgggcactaaggcactggctggtcaatggctggcttacggtgttactcgcagtgtgactaagcgttcagtcatgacgctggcttacgggtccaaagagttcggcttccgtcaacaagtgctggaagataccattcagccagctattgattccggcaagggtctgatgttcactcagccgaatcaggctgctggatacatggctaagctgatttgggaatctgtgagcgtgacggtggtagctgcggttgaagcaatgaactggcttaagtctgctgctaagctgctggctgctgaggtcaaagataagaagactggagagattcttcgcaagcgttgcgctgtgcattgggtaactcctgatggtttccctgtgtggcaggaatacaagaagcctattcagacgcgcttgaacctgatgttcctcggtcagttccgcttacagcctaccattaacaccaacaaagatagcgagattgatgcacacaaacaggagtctggtatcgctcctaactttgtacacagccaagacggtagccaccttcgtaagactgtagtgtgggcacacgagaagtacggaatcgaatcttttgcactgattcacgactccttcggtaccattccggctgacgctgcgaacctgttcaaagcagtgcgcgaaactatggttgacacatatgagtcttgtgatgtactggctgatttctacgaccagttcgctgaccagttgcacgagtctcaattggacaaaatgccagcacttccggctaaaggtaacttgaacctccgtgacatcttagagtcggacttcgcgttcgcgtaaGCTTccgCTGCAGcccGAGCTCgcttcctggtgtccctgttgataccgggaagccctgggccaacttttggcgaaaatgagacgttgatcggcacgtaagaggttccaactttcaccataatgaaataagatcactaccgggcgtattttttgagttatcgagattttcaggagctaaggaagctaaaATGgagaaaaaaatcactggatataccaccgttgatatatcccaatggcatcgtaaagaacattttgaggcatttcagtcagttgctcaatgtacctataaccagaccgttcagctggatattacggcctttttaaagaccgtaaagaaaaataagcacaagttttatccggcctttattcacattcttgcccgcctgatgaatgctcatccggaattccgtatggcaatgaaagacggtgagctggtgatatgggatagtgttcacccttgttacaccgttttccatgagcaaactgaaacgttttcatcgctctggagtgaataccacgacgatttccggcagtttctacacatatattcgcaagatgtggcgtgttacggtgaaaacctggcctatttccctaaagggtttattgagaatatgtttttcgtctcagccaatccctgggtgagtttcaccagttttgatttaaacgtggccaatatggacaacttcttcgcccccgttttcaccatgggcaaatattatacgcaaggcgacaaggtgctgatgccgctggcgattcaggttcatcatgccgtctgtgatggcttccatgtcggcagaatgcttaatgaattacaacagtactgcgatgagtggcagggcggggcgTAAtttttttaaggcagttattggtgcccttaaacgcctggtGTAAAAACCCGCTTCGGCGGGTTTTTTTATGctgattaagcattggtaactgtcagaccaagtttactcatatatactttagattgatttaaaacttcatttttaatttaaaaggatctaggtgaagatcctttttgataatctcatgaccaaaatcccttaacgtgagttttcgttccactgagcgtcagaccccttaataagatgatcttcttgagatcgttttggtctgcgcgtaatctcttgctctgaaaacgaaaaaaccgccttgcagggcggtttttcgaaggttctctgagctaccaactctttgaaccgaggtaactggcttggaggagcgcagtcaccaaaacttgtcctttcagtttagccttaaccggcgcatgacttcaagactaactcctctaaatcaattaccagtggctgctgccagtggtgcttttgcatgtctttccgggttggactcaagacgatagttaccggataaggcgcagcggtcggactgaacggggggttcgtgcatacagtccagcttggagcgaactgcctacccggaactgagtgtcaggcgtggaatgagacaaacgcggccataacagcggaatgacaccggtaaaccgaaaggcaggaacaggagagcgcacgagggagccgccagggggaaacgcctggtatctttatagtcctgtcgggtttcgccaccactgatttgagcgtcagatttcgtgatgcttgtcaggggggcggagcctatggaaaaacggctttgccgcggccctctcacttccctgttaagtatcttcctggcatcttccaggaaatctccgccccgttcgtaagccatttccgctcgccgcagtcgaacgaccgagcgtagcgagtcagtgagcgaggaagcggaatatatcctgtatcacatattctgctgacgcaccggtgcagccttttttctcctgccacatgaagcacttcactgacaccctcatcagtgccaacatagtaagccagtatacactccgctagcgctgaggt

**pJG1113**

*msfGFP* is located in position 1321-2037 bp

*mScarlet-I* is located in position 2107-2802 bp

Gene that confers Ampicillin resistance is located at position 2933-3793 bp

pCDF *ori* is located in 3866-4604 bp

*lacI* is located at position 59-1141 bp

CTCGAGgatcgatcccggtgcctaatgagtgagctaacttacattaattgcgttgcgcTCActgcccgctttccagtcgggaaacctgtcgtgccagctgcattaatgaatcggccaacgcgcggggagaggcggtttgcgtattgggcgccagggtggtttttcttttcaccagtgagacgggcaacagctgattgcccttcaccgcctggccctgagagagttgcagcaagcggtccacgctggtttgccccagcaggcgaaaatcctgtttgatggtggttaacggcgggatataacatgagctgtcttcggtatcgtcgtatcccactaccgagatgtccgcaccaacgcgcagcccggactcggtaatggcgcgcattgcgcccagcgccatctgatcgttggcaaccagcatcgcagtgggaacgatgccctcattcagcatttgcatggtttgttgaaaaccggacatggcactccagtcgccttcccgttccgctatcggctgaatttgattgcgagtgagatatttatgccagccagccagacgcagacgcgccgagacagaacttaatgggcccgctaacagcgcgatttgctggtgacccaatgcgaccagatgctccacgcccagtcgcgtaccgtcttcatgggagaaaataatactgttgatgggtgtctggtcagagacatcaagaaataacgccggaacattagtgcaggcagcttccacagcaatggcatcctggtcatccagcggatagttaatgatcagcccactgacgcgttgcgcgagaagattgtgcaccgccgctttacaggcttcgacgccgcttcgttctaccatcgacaccaccacgctggcacccagttgatcggcgcgagatttaatcgccgcgacaatttgcgacggcgcgtgcagggccagactggaggtggcaacgccaatcagcaacgactgtttgcccgccagttgttgtgccacgcggttgggaatgtaattcagctccgccatcgccgcttccactttttcccgcgttttcgcagaaacgtggctggcctggttcaccacgcgggaaacggtctgataagagacaccggcatactctgcgacatcgtataacgttactggtttCACattcaccaccctgaattgactctcttccgggcgctATCATGccataccgcgaaaggttTTGCGCcattcgatggtgtccgggaATtcTAATACGACTCACTATAGggAATTGTGAGCGCTCACAATTccacaacggtTCTAGAaataattttgtttaactttaagaaggagatataCATatgAGCAAGGGCGAGGAGCTGTTCACCGGGGTGGTGCCCATCCTGGTCGAGCTGGACGGCGACGTAAACGGCCACAAGTTCAGCGTGCGCGGCGAGGGCGAGGGCGATGCCACCAACGGCAAGCTGACCCTGAAGTTCATCTGCACCACCGGCAAGCTGCCCGTGCCCTGGCCCACCCTCGTGACCACCCTGACCTACGGCGTGCAGTGCTTCAGCCGCTACCCCGACCACATGAAGCAGCACGACTTCTTCAAGTCCGCCATGCCCGAAGGCTACGTCCAGGAGCGCACCATCTCCTTCAAGGACGACGGCACCTACAAGACCCGCGCCGAGGTGAAGTTCGAGGGCGACACCCTGGTGAACCGCATCGAGCTGAAGGGCATCGACTTCAAGGAGGACGGCAACATCCTGGGGCACAAGCTGGAGTACAACTTCAACAGCCACAACGTCTATATCACGGCCGACAAGCAGAAGAACGGCATCAAGGCGAACTTCAAGATCCGCCACAACGTCGAGGACGGCAGCGTGCAGCTCGCCGACCACTACCAGCAGAACACCCCCATCGGCGACGGCCCCGTGCTGCTGCCCGACAACCACTACCTGAGCACCCAGTCCAAGCTGAGCAAAGACCCCAACGAGAAGCGCGATCACATGGTCCTGCTGGAGTTCGTGACCGCCGCCGGGATCACTCTCGGCATGGACGAGCTGTACAAGtgacgctgagcagtaactaGGATCCacaGGTACCgaaataattttgtttaactttaagaaggagatatacatATGGTGAGCAAGGGCGAGGCAGTGATCAAGGAGTTCATGCGGTTCAAGGTGCACATGGAGGGCTCCATGAACGGCCACGAGTTCGAGATCGAGGGCGAGGGCGAGGGCCGCCCCTACGAGGGCACCCAGACCGCCAAGCTGAAGGTGACCAAGGGTGGCCCCCTGCCCTTCTCCTGGGACATCCTGTCCCCTCAGTTCATGTACGGCTCCAGGGCCTTCATCAAGCACCCCGCCGACATCCCCGACTACTATAAGCAGTCCTTCCCCGAGGGCTTCAAGTGGGAGCGCGTGATGAACTTCGAGGACGGCGGCGCCGTGACCGTGACCCAGGACACCTCCCTGGAGGACGGCACCCTGATCTACAAGGTGAAGCTCCGCGGCACCAACTTCCCTCCTGACGGCCCCGTAATGCAGAAGAAGACAATGGGCTGGGAAGCGTCCACCGAGCGGTTGTACCCCGAGGACGGCGTGCTGAAGGGCGACATTAAGATGGCCCTGCGCCTGAAGGACGGCGGCCGCTACCTGGCGGACTTCAAGACCACCTACAAGGCCAAGAAGCCCGTGCAGATGCCCGGCGCCTACAACGTCGACCGCAAGTTGGACATCACCTCCCACAACGAGGACTACACCGTGGTGGAACAGTACGAACGCTCCGAGGGCCGCCACTCCACCGGCGGCATGGACGAGCTGTACAAGtaaGCTTCCcgagCTCggaaatgtgcgcggaacccctatttgtttatttttctaaatacattcaaatatgtatccgctcatgagacaataaccctgataaatgcttcaataatattgaaaaaggaagagtATGagtattcaacatttccgtgtcgcccttattcccttttttgcggcattttgccttcctgtttttgctcacccagaaacgctggtgaaagtaaaagatgctgaagatcagttgggtgcacgagtgggttacatcgaactggatctcaacagcggtaagatccttgagagttttcgccccgaagaacgttttccaatgatgagcacttttaaagttctgctatgtggcgcggtattatcccgtattgacgccgggcaagagcaactcggtcgccgcatacactattctcagaatgacttggttgagtactcaccagtcacagaaaagcatcttacggatggcatgacagtaagagaattatgcagtgctgccataaccatgagtgataacactgcggccaacttacttctgacaacgatcggaggaccgaaggagctaaccgcttttttgcacaacatgggggatcatgtaactcgccttgatcgttgggaaccggagctgaatgaagccataccaaacgacgagcgtgacaccacgatgcctgtagcaatggcaacaacgttgcgcaaactattaactggcgaactacttactctagcttcccggcaacaattaatagactggatggaggcggataaagttgcaggaccacttctgcgctcggcccttccggctggctggtttattgctgataaatctggagccggtgagcgtgggtctcgcggtatcattgcagcactggggccagatggtaagccctcccgtatcgtagttatctacacgacggggagtcaggcaactatggatgaacgaaatagacagatcgctgagataggtgcctcactgattaagcattggTAActgtcagaccaagtttactcGTAAAAACCCGCTTCGGCGGGTTTTTTTATGctagggcggttcagtagaaaagatcaaaggatcttcttgagatcctttttttctgcgcgtaatcttttgccctgtaaacgaaaaaaccacctggggaggtggtttgatcgaaggttaagtcagttggggaactgcttaaccgtggtaactggctttcgcagagcacagcaaccaaatctgtccttccagtgtagccggactttggcgcacacttcaagagcaaccgcgtgtttagctaaacaaatcctctgcgaactcccagttaccaatggctgctgccagtggcgttttaccgtgcttttccgggttggactcaagtgaacagttaccggataaggcgcagcagtcgggctgaacggggagttcttgcttacagcccagcttggagcgaacgacctacaccgagccgagataccagtgtgtgagctatgagaaagcgccacacttcccgtaagggagaaaggcggaacaggtatccggtaaacggcagggtcggaacaggagagcgcaagagggagcgacccgccggaaacggtggggatctttaagtcctgtcgggtttcgcccgtactgtcagattcatggttgagcctcacggctcccacagatgcaccggaaaagcgtctgtttatgtgaactctggcaggagggcggagcctatggaaaaacgccaccggcgcggccctgctgttttgcctcacatgttagtcccctgcttatccacggaatctgtgggtaactttgtatgtgtccgcagcgcccgccgcagtctcacgcccggagcgtagcgaccgagtgaa

**pJG1118**

*msfGFP* is located in position 1356-2072 bp

*mScarlet-I* is located in position 2142-2837 bp

Gene that confers Ampicillin resistance is located at position 2968-3828 bp

pCDF *ori* is located in 3901-4639 bp

*lacI* is located at position 59-1141 bp

CTCGAGgatcgatcccggtgcctaatgagtgagctaacttacattaattgcgttgcgcTCActgcccgctttccagtcgggaaacctgtcgtgccagctgcattaatgaatcggccaacgcgcggggagaggcggtttgcgtattgggcgccagggtggtttttcttttcaccagtgagacgggcaacagctgattgcccttcaccgcctggccctgagagagttgcagcaagcggtccacgctggtttgccccagcaggcgaaaatcctgtttgatggtggttaacggcgggatataacatgagctgtcttcggtatcgtcgtatcccactaccgagatgtccgcaccaacgcgcagcccggactcggtaatggcgcgcattgcgcccagcgccatctgatcgttggcaaccagcatcgcagtgggaacgatgccctcattcagcatttgcatggtttgttgaaaaccggacatggcactccagtcgccttcccgttccgctatcggctgaatttgattgcgagtgagatatttatgccagccagccagacgcagacgcgccgagacagaacttaatgggcccgctaacagcgcgatttgctggtgacccaatgcgaccagatgctccacgcccagtcgcgtaccgtcttcatgggagaaaataatactgttgatgggtgtctggtcagagacatcaagaaataacgccggaacattagtgcaggcagcttccacagcaatggcatcctggtcatccagcggatagttaatgatcagcccactgacgcgttgcgcgagaagattgtgcaccgccgctttacaggcttcgacgccgcttcgttctaccatcgacaccaccacgctggcacccagttgatcggcgcgagatttaatcgccgcgacaatttgcgacggcgcgtgcagggccagactggaggtggcaacgccaatcagcaacgactgtttgcccgccagttgttgtgccacgcggttgggaatgtaattcagctccgccatcgccgcttccactttttcccgcgttttcgcagaaacgtggctggcctggttcaccacgcgggaaacggtctgataagagacaccggcatactctgcgacatcgtataacgttactggtttCACattcaccaccctgaattgactctcttccgggcgctATCATGccataccgcgaaaggttTTGCGCcattcgatggtgtccgggaAttgtgagCGctcacaaTTtcatgaaaaatttatTTGCTTtgtgagcggataacaatTATAATatgtggAAttgtgagcGgataacaaTTctAGAaataattttgtttaactttaagaaggagatataCATatgAGCAAGGGCGAGGAGCTGTTCACCGGGGTGGTGCCCATCCTGGTCGAGCTGGACGGCGACGTAAACGGCCACAAGTTCAGCGTGCGCGGCGAGGGCGAGGGCGATGCCACCAACGGCAAGCTGACCCTGAAGTTCATCTGCACCACCGGCAAGCTGCCCGTGCCCTGGCCCACCCTCGTGACCACCCTGACCTACGGCGTGCAGTGCTTCAGCCGCTACCCCGACCACATGAAGCAGCACGACTTCTTCAAGTCCGCCATGCCCGAAGGCTACGTCCAGGAGCGCACCATCTCCTTCAAGGACGACGGCACCTACAAGACCCGCGCCGAGGTGAAGTTCGAGGGCGACACCCTGGTGAACCGCATCGAGCTGAAGGGCATCGACTTCAAGGAGGACGGCAACATCCTGGGGCACAAGCTGGAGTACAACTTCAACAGCCACAACGTCTATATCACGGCCGACAAGCAGAAGAACGGCATCAAGGCGAACTTCAAGATCCGCCACAACGTCGAGGACGGCAGCGTGCAGCTCGCCGACCACTACCAGCAGAACACCCCCATCGGCGACGGCCCCGTGCTGCTGCCCGACAACCACTACCTGAGCACCCAGTCCAAGCTGAGCAAAGACCCCAACGAGAAGCGCGATCACATGGTCCTGCTGGAGTTCGTGACCGCCGCCGGGATCACTCTCGGCATGGACGAGCTGTACAAGtgacgctgagcagtaactaGGATCCacaGGTACCgaaataattttgtttaactttaagaaggagatatacatATGGTGAGCAAGGGCGAGGCAGTGATCAAGGAGTTCATGCGGTTCAAGGTGCACATGGAGGGCTCCATGAACGGCCACGAGTTCGAGATCGAGGGCGAGGGCGAGGGCCGCCCCTACGAGGGCACCCAGACCGCCAAGCTGAAGGTGACCAAGGGTGGCCCCCTGCCCTTCTCCTGGGACATCCTGTCCCCTCAGTTCATGTACGGCTCCAGGGCCTTCATCAAGCACCCCGCCGACATCCCCGACTACTATAAGCAGTCCTTCCCCGAGGGCTTCAAGTGGGAGCGCGTGATGAACTTCGAGGACGGCGGCGCCGTGACCGTGACCCAGGACACCTCCCTGGAGGACGGCACCCTGATCTACAAGGTGAAGCTCCGCGGCACCAACTTCCCTCCTGACGGCCCCGTAATGCAGAAGAAGACAATGGGCTGGGAAGCGTCCACCGAGCGGTTGTACCCCGAGGACGGCGTGCTGAAGGGCGACATTAAGATGGCCCTGCGCCTGAAGGACGGCGGCCGCTACCTGGCGGACTTCAAGACCACCTACAAGGCCAAGAAGCCCGTGCAGATGCCCGGCGCCTACAACGTCGACCGCAAGTTGGACATCACCTCCCACAACGAGGACTACACCGTGGTGGAACAGTACGAACGCTCCGAGGGCCGCCACTCCACCGGCGGCATGGACGAGCTGTACAAGtaaGCTTCCcgagCTCggaaatgtgcgcggaacccctatttgtttatttttctaaatacattcaaatatgtatccgctcatgagacaataaccctgataaatgcttcaataatattgaaaaaggaagagtATGagtattcaacatttccgtgtcgcccttattcccttttttgcggcattttgccttcctgtttttgctcacccagaaacgctggtgaaagtaaaagatgctgaagatcagttgggtgcacgagtgggttacatcgaactggatctcaacagcggtaagatccttgagagttttcgccccgaagaacgttttccaatgatgagcacttttaaagttctgctatgtggcgcggtattatcccgtattgacgccgggcaagagcaactcggtcgccgcatacactattctcagaatgacttggttgagtactcaccagtcacagaaaagcatcttacggatggcatgacagtaagagaattatgcagtgctgccataaccatgagtgataacactgcggccaacttacttctgacaacgatcggaggaccgaaggagctaaccgcttttttgcacaacatgggggatcatgtaactcgccttgatcgttgggaaccggagctgaatgaagccataccaaacgacgagcgtgacaccacgatgcctgtagcaatggcaacaacgttgcgcaaactattaactggcgaactacttactctagcttcccggcaacaattaatagactggatggaggcggataaagttgcaggaccacttctgcgctcggcccttccggctggctggtttattgctgataaatctggagccggtgagcgtgggtctcgcggtatcattgcagcactggggccagatggtaagccctcccgtatcgtagttatctacacgacggggagtcaggcaactatggatgaacgaaatagacagatcgctgagataggtgcctcactgattaagcattggTAActgtcagaccaagtttactcGTAAAAACCCGCTTCGGCGGGTTTTTTTATGctagggcggttcagtagaaaagatcaaaggatcttcttgagatcctttttttctgcgcgtaatcttttgccctgtaaacgaaaaaaccacctggggaggtggtttgatcgaaggttaagtcagttggggaactgcttaaccgtggtaactggctttcgcagagcacagcaaccaaatctgtccttccagtgtagccggactttggcgcacacttcaagagcaaccgcgtgtttagctaaacaaatcctctgcgaactcccagttaccaatggctgctgccagtggcgttttaccgtgcttttccgggttggactcaagtgaacagttaccggataaggcgcagcagtcgggctgaacggggagttcttgcttacagcccagcttggagcgaacgacctacaccgagccgagataccagtgtgtgagctatgagaaagcgccacacttcccgtaagggagaaaggcggaacaggtatccggtaaacggcagggtcggaacaggagagcgcaagagggagcgacccgccggaaacggtggggatctttaagtcctgtcgggtttcgcccgtactgtcagattcatggttgagcctcacggctcccacagatgcaccggaaaagcgtctgtttatgtgaactctggcaggagggcggagcctatggaaaaacgccaccggcgcggccctgctgttttgcctcacatgttagtcccctgcttatccacggaatctgtgggtaactttgtatgtgtccgcagcgcccgccgcagtctcacgcccggagcgtagcgaccgagtgaa
